# Approaches for Effective Clinical Application of Stem Cell Transplantation

**DOI:** 10.1007/s40472-018-0202-0

**Published:** 2018-08-02

**Authors:** E. Attico, V. Sceberras, G. Pellegrini

**Affiliations:** 1 Centre for Regenerative Medicine “Stefano Ferrari”, University of Modena and Reggio Emilia, Modena, Italy; 2 Holostem Terapie Avanzate, Modena, Italy

**Keywords:** ATMP, Translational medicine, Stem cell, Gene therapy, Cell therapy, Clinical trial, Valley of death

## Abstract

**Purpose of Review:**

This review highlights problems related to translation of advanced therapy medicinal products (ATMPs) from bench to bedsite. Regenerative medicine within the current regulatory frame reveals common hitches in the course of development, translation, and clinical application. This paper suggests outlining a path from the few examples of successfully approved vs unsuccessful advanced therapies.

**Recent Findings:**

In the multitude of ongoing studies, few of them achieved positive results with a final treatment available to patients; this result was possible due to multidisciplinary teams working together from the beginning of the development and during the hard route to standardization and clinical application.

**Summary:**

The root of success of an advanced therapy requires not only the inescapable scientific and biological knowledge but also requires several contributions as regulatory, ethical, medical, and bio-engineering expertise, from the real beginning. A strong scientific rationale and an integrated network of expertises would contribute to a successful investment of available resources in advanced therapy medicinal products and to a greater confidence in future medicine.

## Introduction

Stem cell transplantation has raised great expectations for fulfilling unmet medical needs, and substantial resources have been invested to explore stem cell applications and develop novel therapies. Despite the impressive technological evolution, only a limited percentage of these studies has reached clinical application and only few of them have obtained positive results [1, 2, 3•, 4•].

The resulting picture is a technology with great potential that is being viewed with skepticism and a progressive lack of confidence.

The analysis of failures and the related “valley of death”, which is also described for chemical drugs, has produced a biased description of causes: scientists ascribe setbacks to technicalities and shortage of funding, entrepreneurs blame scientists for insufficient long-term planning and lack of data reproducibility, regulators criticize the lack of accuracy in quality or safety evaluations, patients complain of the absence of therapies for severe diseases, and governments bemoan the increasing health costs. What is the real problem? Is it the sum of all of the above?

Most root cause analyses are biased since they describe a single perspective, revealing that cross-fertilization is missing, even though many interdisciplinary meetings have been conducted. Inappropriate communication between different professionals having their own priorities and technical languages has been frequently observed.

**Fig. 1:**
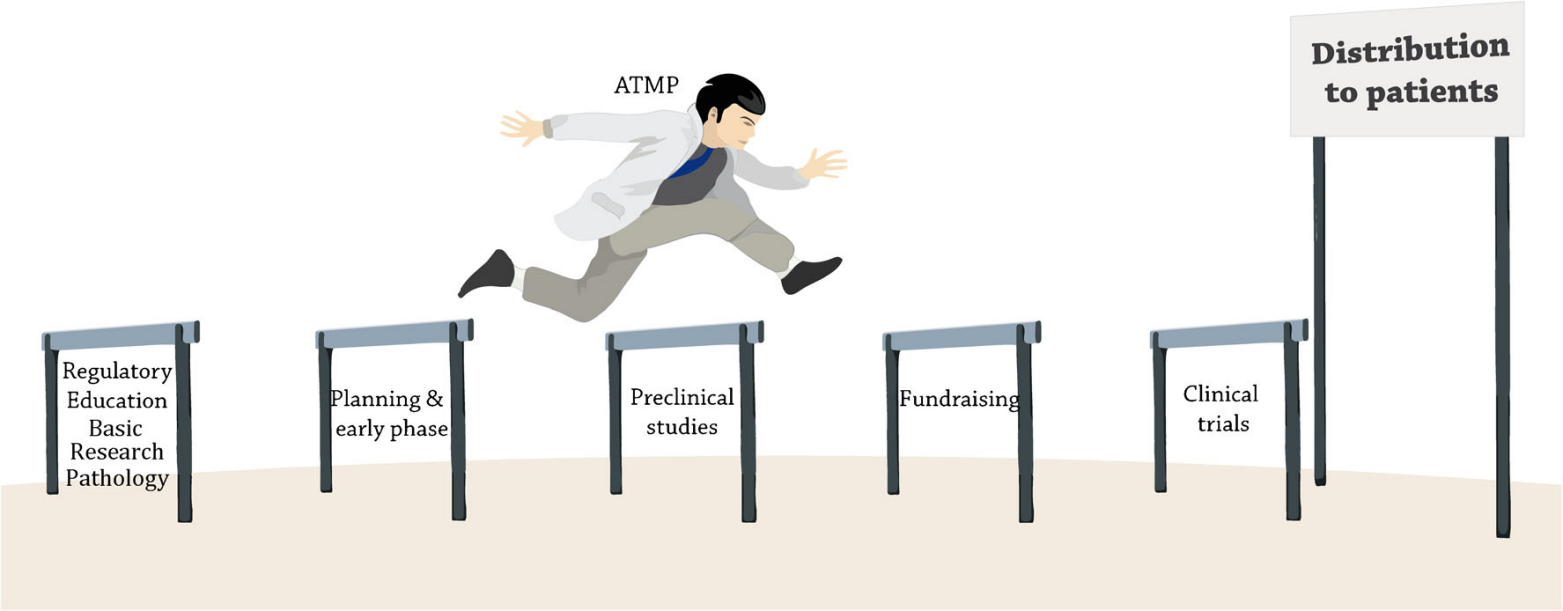
Translation of ATMP from bench to bedsite. The different phases of development for a new stem cell therapy

It is clear that specific experts hold leading positions in different phases of development for a new stem cell therapy: scientists in the early phase of research; physicians in clinical trials; and entrepreneurs in the evaluation of opportunities, organization, and funding; as well as public payers and regulators in the reviewing of preclinical and clinical data. They are all involved in the translation and forming the “tower of Babel” and contributing to the valley of death (Fig. 1).

From a scientific viewpoint, research is usually conducted to define the experimental condition and observe/understand cell behavior. Shifting this attitude toward controlling the conditions in order to develop a specific product is not easy. This difference can produce variability and problems in standardization. Taking control of manufacturing conditions requires a very deep knowledge of the process, and the pressure to “publish or perish” can result in inaccurate or fake research. In addition, scientific journals primarily publish positive results, and failures are rarely reported. Further, data are usually described in a limited number of words, not listing all the technical details critical for product reproducibility.

In case of cell therapies that often involve extensive cell manipulation, the regulatory requirements should be considered from the real beginning (planning) of research. The definition of experimental conditions for therapeutic development should only include materials with a “clinical level” safety profile, and should, therefore, be produced by organisms or processes in which disease transmission, toxicity, or impurities could be controlled. Tissues should be manufactured reproducibly and possibly reagents should not leave detectable residues in the final cell preparation administered to patients.

The number of replicates produced for scientific papers are usually insufficient for clinical translation, where a quantitative description of all parameters is required despite the possible high biological variability in the general population.

In a typical case, if the expression of a specific cell parameter increases by 30% in the culture phase, the lower and upper limit of expression should be defined in cell therapy protocols intended for clinical application; a standard question could be 28–32% or 10–40%? How was the interval defined? How to validate the assays for detection and quantification? What is a possible reference standard for the marker in the validation assay?

The answers to these questions require a large number of replicates to balance biological variability defining the limits applicable to many different individuals and considering a “safety margin.”

Since the complete exercise is not easy, successful examples of regulatory approval and transplantation for both cell and gene therapies should provide a track for other future therapies.

Here, we report some examples from the developmental phases of successful and failed tissue engineering products, i.e., Holoclar® [1] and tracheal reconstruction [5], and two successful examples of gene therapy, i.e., a registered product (Strimvelis®) [3•] for the treatment of ADA deficiency and a phase I/II trial for the treatment of junctional epidermolysis bullosa [6].

## Early Phase and Planning

In Europe, stem cell cultures are considered as advanced therapy medicinal products. Therefore, they need to be compliant with (European Medicinal Agency) cGMP rules. The GMP rules cannot include anything that is not validated or not produced following the “quality” criteria. Cell cultures including stem cells may need many different components, such as serum (of animal or human origin) or simply culture medium and growth factors that are not always produced for human use. On the same note, most of these components are not included in the pharmacopeia. Therefore, no “official” assay for their control is envisaged and it becomes a scientist’s role to propose a safe origin for each component, GMP-compliant assays, and a reference standard for their control.

Thus, knowledge of these rules is mandatory for the scientist from the beginning of the research.

This further implies that the development of these therapies should foresee the training of biologists on regulatory rules, from the beginning of their education (or at least before developing the therapies).

This is not commonly done, leading to development of putative therapies, which need to be completely redefined later, with an increase in failures, time, and costs.

The types of controls on raw materials for advanced therapy planning should be based not only on the risks related to contaminants and impurities but also on their capacity to maintain biological functions relevant to the foreseen mechanism of action of the product. In the case of stem cell products, it means that no reagent should interfere with the ability of these specific cells to form a tissue and to maintain their capacities over their lifetime. In the case of advanced therapies approved at the European level, “in-process controls” measuring the expression of markers/properties validated for their correlation with stem cell-specific functions, such as proliferative potential in vitro and successful in vivo regeneration properties, are envisaged. These are different from the assessment of active proliferation as they are a measure of the capacity to maintain proliferation over the lifetime of the culture and in vivo. In the case of gene therapy [7], long-term expression of the transgene that is missing or defective in the pathology should also be included as an in-process control.

In the case of the proposed examples of advanced therapy medicinal products, the “regulatory controls” on raw materials were not in place in the early phase of the research, as they were developed before the ATMP regulation was passed in Europe. However, it should be noted that a significant number of basic research investigations were conducted for three of these ATMPs, namely Holoclar®, Strimvelis®, and junctional epidermolysis bullosa treatment before their clinical application, and many investigations were also conducted on the cell identification and their specific properties [8•, 9•, 10].

In the case of tracheal reconstruction, these studies appeared insufficient for a reproducible and efficient ATMP.

One critical issue for cell therapy development is the potency assay, requested by regulatory authorities and defined in the ICH Q6B guideline [11] as a “measure of the biological activity using a suitable quantitative biological assay, based on the attribute of the product which is linked to the relevant biological properties”. This request comes from the past when elucidation of drug structure was limited and the measure of “content” was requested as quantification of biological activity related to structural integrity [12]. In stem cell therapy as well as for all ATMPs, biological activity can involve multiple pathways, and therefore, scientists should identify a “tailored” assay including activities possibly related to function. In other words, a relationship between the results of selected assays and their clinical effect (efficacy/safety) is expected. This prospect implies that the scientist would know what and how much a specific cell function is responsible for the mechanism of action of the therapy.

This knowledge is usually confirmed after clinical application, when the mechanism of action is fully understood. Once the potency assay is identified, it becomes instrumental for the evaluation of the drug/therapy, the manufacturing process changes, the release approval, the stability, and so on. Indeed, any alteration in cell performance could be easily highlighted by a comparative measure of potency, allowing appropriate selection of conditions and control of their maintenance.

The registered stem cell product, Holoclar®, identified a specific stem cell marker, p63, used as a potency assay since its mechanism was to replace lost stem cells [8•, 13]. The gene therapy products (Strimvelis®) and the epidermolysis bullosa treatment proposed the percentage of transduction with the transgene associated with the quantitative measure of capacity for tissue regeneration: CD34 and colony forming efficiency, respectively [6, 14]. No potency marker is known for the tracheal reconstruction.

In the timeline, all in-process controls are defined and run early in the process development. Therefore, functions and marker maintenance as well as their correlation with in vivo behavior need to be identified well before their clinical application, in adequate models for product testing.

This implies that a significant number of basic research investigations should be conducted before planning the clinical application of any specific model.

## Preclinical Study

The preclinical phase can be run on relevant animal models if any, integrated with in vitro prototypes, or using in vitro systems only. The use of autologous cultured animal cell transplantation cannot provide many safety indications, as frequently animals present substantial differences in stem cell culture conditions, microenvironment, and distribution with respect to human tissues [15, 16]. In addition, the size of the tissue is frequently different and is related to the cell dosage and microenvironment. Use of this approach would lead to the need of repetition for all settings of the therapy in human conditions with the loss of time and costs.

The problem with animal models is a significant issue for stem cell products because in parallel to high costs, xenotransplantation can result in poor engraftment where the safety of the treatment (due to in vivo cell behavior) is not properly highlighted.

Typically, in vivo models are used for evaluating the biodistribution of transplanted cells and the analysis of uncontrolled cell growth due to improper extensive manipulation. For both, integration of cells in the model is mandatory for safety evaluation.

It was proven that the repair of some very severe lesions was due to the engraftment of cultured cells and not to “circulating” cells. This was coherently shown in gene therapy treatments where autologous cells are genetically different from the resident cells and are found to repopulate the entire tissue after culture and transplant [4•]. Examples include ADA-deficiency treatment with genetically modified CD34+ blood cells or junctional epidermolysis bullosa therapy with LM5-beta 3-transduced epidermal cells, after approximately 10 years of follow-up in both cases [6, 14, 17].

Indeed, animal models would have no meaning for safety evaluation without a proven engraftment of transplanted cells.

The tissue physiology in different animal species can be an obstacle for understanding human stem cell behavior. In the case of at least two gene therapy protocols, the absence of resident stem cells was shown as a pre-requisite for transduced cell engraftment and efficacy. This also holds true for other safety evaluations, where transplanted stem cell tumorigenicity cannot be evaluated properly, in the presence of mixed human and animal cells producing a variable rate of integration and a different microenvironment. The experience with the epidermolysis bullosa treatment revealed an absence of migration in the integrated, GFP-labeled human cells in the animal model [18]. Conversely, the model failed to reproduce human cell integration for Holoclar (data not shown), and the published data on trachea did not report the dramatic failures shown in humans, when the tissue was tested in animals [19]. In the case of Strimvelis® where the migration occurs in the physiology of hematopoietic tissues, the absence of uncontrolled proliferation was proven [20–22]. Thus, the use of human in vitro models is probably desirable for a realistic evaluation of any therapy.

On this line, it is necessary to make specific distinctions when using embryonic or induced pluripotent stem cells. These cells are committed to differentiation into specific tissues before in vivo transfer; when few uncommitted cells persist, in vivo models can highlight the development of teratomas, which could be not so evident in vitro*.*

On the other hand, commitment should not be driven up to somatic stem cell loss in order to preserve tissue self-renewal. This issue can be easily highlighted and characterized by integration with in vitro models. Finally, the additional use of human in vitro models has several advantages such as increasing the knowledge of tested ATMPs, the biological behavior of the tissue, and not least, the cost optimization before clinical application.

## Clinical Application

A specific clinical question is usually defined at the real beginning of the study. This is one of the most important phases of a study, as poorly focused questions lead to unclear decisions.

The study of clinical applications allows the definition of formulations compatible with putative surgery, mode of administration, and patient selection. Identification of a real unmet medical need should drive meaningful research through which patients and health care professionals bring in new insights with respect to research priorities, treatment, and outcomes. All relevant parties should be involved in this phase defining the clinical questions, developing the trial protocol, and commenting on the preliminary design.

The evaluation is related to risk/benefit ratio as well as to whether a medicine is cost effective in relation to other treatment alternatives, answered in the social context.

For low levels of severity in different pathologies, treatments are frequently available but few or no alternatives are in place for the highest severity score. This implies that the selection of patients as well as the definition of ATMP clinical outcomes should be indicated for a specific threshold of inclemency. Indeed, patient group differences, in particular, the differential baseline risk of the condition and the capacity of different subgroups of patients to benefit, must be considered.

One example comes from cartilage repair. There are different treatment options for cartilage defects, depending on defect size and the state of the subchondral bone. Cartilage defects can be treated by surgery, bone marrow-stimulating techniques, osteochondral autograft transplantation, or implantation of cells and/or scaffolds. Autologous chondrocyte implantation (ACI) and scaffold-supported ACI are often used for treating large chondral defects (> 2 cm^2^), where other treatment options would be ineffective [23].

Clinical trials for ATMPs should include almost exclusively severe lesions for which repair is more challenging than for mild lesions. However, the consequence is a lower expectation for the related clinical outcome in a cost/benefit ratio evaluation. This applies (as an example) to urethral stenosis: some forms of these have a high success rate with surgery, but others have a very high failure rate due to lichen sclerosus or failure after hypospadia treatment, resulting in disability and requiring new therapeutic approaches [24].

In the case of Holoclar®, Strimvelis®, and the phase I/II junctional epidermolysis bullosa treatment, the patient selection criteria were very well-defined and selection was based on either a precise definition of pathology associated to an accurate grading or a genetic diagnosis of the affected individuals [6, 8•, 14]. The tracheal pathologies highlighted a significant unmet medical need for reconstruction but the applied selection of patients was too wide and included many different conditions [25].

When the outcomes of clinical and pharmaco-economic analyses are not properly defined or not considered by clinicians or scientists, problems are raised at a later stage by regulators and payers.

Concerning medicine formulation, severely damaged wound beds require different surgery as well as different stem cell products, i.e., the presence of carrier, substrate or cells only, whole tissue or layers of cells, enrichment of stem cells, or a defined ratio between stem and differentiated cells. Different therapeutic formulations could apply to the same tissue, depending on the pathology, surgery, and the resulting wound bed, and requires an accurate analysis. For example, epithelial cells from the cornea are usually cultured and transplanted with a carrier due to the fragility of the cultured corneal epithelium [26], whereas epithelium from the airway was cultured on a scaffold to reconstitute the trachea [27]. Vitiligo treatments have been proposed using cells (melanocytes) or epithelium only, due to the need for rapid engraftment to superficial lesions [28]. Epithelia are usually transplanted with a defined proportion of stem and differentiated cells to quickly provide a barrier and to maintain the normal tissue homeostasis. In the case of bone marrow, a stem cell-enriched fraction is transplanted, since the mechanism of action envisages a rapid recolonization of the niche [29]. All these different choices have implications on cell therapy development together with an accurate testing of long-term compatibility of putative carriers and cell/scaffold interaction with ancillary drugs administered to the specific selection of patients. An optimal design should be considered, even for effectiveness needs, and activities must be implemented in the early stage of research, through collaboration of biologists and clinicians, as in previously mentioned successful therapies.

## Additional Issues

It is difficult to complete the list of hurdles preventing the efficient translation of ATMPs. However, some additional issues need to be considered. Two of these include the training of clinical teams on GMP rules and the administration of ATMPs. The GMP rules in force for ATMPs require well-defined procedures for all activities and extensive reporting. Specific training is thus needed for the clinical team in order to enable physicians, surgeons, and nurses to properly manage all the requested documents. The issue of ATMP administration and handling requires a specific knowledge of the product, its fragility, and the limitations related to the use of an in vitro-assembled, actively proliferating tissue, which is different from the standard whole-tissue transplant. To guarantee efficient translation, the presence of a medical service providing knowledge on the product specificities is desirable both from the viewpoint of regulators and the clinical team. This approach was followed for Holoclar® and the epidermolysis bullosa products, and specific training was provided to a single team managing the therapy for Strimvelis® and for tracheal reconstruction.

Finally, the problem of cost containment must be considered. The choice between organizations under hospital exemption or company fell on a mixed regime for three of the selected examples. Holoclar®, Strimvelis®, and the junctional epidermolysis bullosa treatment began their research and in-depth investigations on the product in a free research environment under hospital exemption and opted for a company setting at the time of registration and distribution, when industrial knowledge was essential to ensure widespread use of the technologies. Trachea reconstruction was always performed under hospital exemption, but never achieved a wide distribution.

Undoubtedly, cost minimization will change dramatically when the entire planning of ATMP development will not need duplication, as was seen before/after the introduction of GMP and regulatory rules. The cost profile will be reduced when surgeons and biologists will begin development by considering the rules. Additional support will be obtained from sharing resources and responsibility, as in the case of private-public partnerships, and by tailored automation and scaling up of production.

## Conclusion

The clinical translation of medically promising cell and gene therapy approaches has been slow and expensive and has shown poor efficiency. In part, clinical translation has been hampered by biased research practices that lead to publication of studies with low reproducibility. These include small, non-predictive studies; improperly designed studies, especially those with biased or undefined clinical experimental groups and lacking necessary controls; studies using uncharacterized or poorly characterized cells and materials, a low number of replicates, or non-validated methods; studies conducted with a poor understanding of the relevant biology and mechanisms; and studies lacking interdisciplinarity. The problems are further compounded by a culture that does not incentivize sharing but encourages poor or selective reporting, the high costs of materials and equipment, and poor flexibility of companies in the application of new approaches to the development of ATMP products. In this review, we discussed some of these problems in light of successful and unsuccessful examples of gene and cell therapy and provided mitigating strategies. Progress on reducing bias, enhancing reproducibility, and investing in multidisciplinary education with an in-depth analysis of processes and pathologies should enhance the translational potential of preclinical technologies.
